# Brain serotonin deficiency affects female aggression

**DOI:** 10.1038/s41598-018-37613-4

**Published:** 2019-02-04

**Authors:** Niklas Kästner, S. Helene Richter, Sarah Urbanik, Joachim Kunert, Jonas Waider, Klaus-Peter Lesch, Sylvia Kaiser, Norbert Sachser

**Affiliations:** 1 Department of Behavioural Biology, University of Münster, Münster, Germany; 2 Münster Graduate School of Evolution, University of Münster, Münster, Germany; 3 Department of Mathematical Statistics and Applications in Science, Technical University of Dortmund, Dortmund, Germany; 4 Division of Molecular Psychiatry, Center of Mental Health, University of Würzburg, Würzburg, Germany; 5 Laboratory of Psychiatric Neurobiology, Institute of Molecular Medicine, I.M. Sechenov First Moscow State Medical University, Moscow, Russia; 6 Department of Neuroscience, School for Mental Health and Neuroscience (MHeNS), Maastricht University, Maastricht, The Netherlands

## Abstract

The neurotransmitter serotonin plays a key role in the control of aggressive behaviour. While so far most studies have investigated variation in serotonin levels, a recently created tryptophan hydroxylase 2 (Tph2) knockout mouse model allows studying effects of complete brain serotonin deficiency. First studies revealed increased aggressiveness in homozygous Tph2 knockout mice in the context of a resident-intruder paradigm. Focussing on females, this study aimed to elucidate effects of serotonin deficiency on aggressive and non-aggressive social behaviours not in a test situation but a natural setting. For this purpose, female Tph2 wildtype (n = 40) and homozygous knockout mice (n = 40) were housed with a same-sex conspecific of either the same or the other genotype in large terraria. The main findings were: knockout females displayed untypically high levels of aggressive behaviour even after several days of co-housing. Notably, in response to aggressive knockout partners, they showed increased levels of defensive behaviours. While most studies on aggression in rodents have focussed on males, this study suggests a significant involvement of serotonin also in the control of female aggression. Future research will show, whether the observed behavioural effects are directly caused by the lack of serotonin or by potential compensatory mechanisms.

## Introduction

Serotonin (5-hydroxy-tryptamin, 5-HT) is a phylogenetically ancient molecule being present in almost every living organism^1^. In mammals, 5-HT affects various behavioural traits, among them mood, emotion and cognition as well as social behaviour^2^. Especially 5-HT’s role in the regulation of aggression has been aim of intensive research^3–6^. First results in mice^7^ and humans^8^ indicated an inverse relation between 5-HT concentrations and aggressiveness, i.e. the lower the 5-HT level, the higher the aggression. This so-called “serotonin deficiency hypothesis”^3,9^ has been supported by further experimental research in rodents: reduced 5-HT concentrations caused by diet, pharmacological interventions or genetic variation increase aggressive behaviour^10–12^, while activation of certain 5-HT receptors as well as genetically increased 5-HT concentrations result in decreased levels of aggressive behaviour^6,13–16^.

While so far most studies in animals have investigated effects of higher vs. lower extracellular 5-HT levels, a tryptophan hydroxylase 2 (Tph2) knockout mouse model offers a new approach. TPH2 is the rate-limiting enzyme in the synthesis of brain 5-HT. Thus, homozygous knockout of the corresponding gene allow to investigate effects of complete brain 5-HT deficiency^17^. Despite the lack of this important neurotransmitter that was confirmed by immunohistochemical analysis, these mice are viable and even develop “serotonergic”-like neurons in the brainstem raphe region^17^. While conflicting effects on anxiety-like and depression-related behaviours have been reported^17–20^, the most prominent result was indeed increased aggressiveness in both sexes^19,21–23^. In addition, compromised social memory and decreased social interest were described^23^.

Yet, so far social behaviour of Tph2 knockout mice has only been assessed in common short tests used for phenotyping. Aggressiveness, for example, has been measured in the context of a resident-intruder paradigm: mice were confronted with a same-sex wildtype conspecific that was introduced into their cage for a duration of 5 or 10 minutes and attacks of the resident towards the intruder were recorded^19,21^.

In contrast to these first investigations, the present study applied a different approach not relying on short test paradigms. We aimed to assess spontaneous social behaviour of wildtype and knockout females in a more natural setting and for a longer duration. Furthermore, we intended to investigate aggressive as well as non-aggressive behaviours towards wildtype as well as knockout individuals. For this purpose, female Tph2 wildtype and knockout mice (focal animals) were housed with an unfamiliar same-sex mouse (partner animal) of either the same or the other genotype. To allow the display of a wide range of behaviours and offer the possibility to avoid each other, the animals were housed in large, enriched terraria^24,25^. Various social behaviours of the focal towards the partner animal were recorded directly after introduction into the terrarium as well as after several days of co-housing based on previous work^5,15,25–29^.

We expected to find a difference in social behaviour between Tph2 knockout and wildtype focal mice, particularly concerning aggressive behaviours, and we expected the combination of genotypes to affect social behaviour. To test these hypotheses, behaviour of the focal animal was analysed for an effect of the focal animal’s genotype, the partner animal’s genotype as well as interactions between these factors.

## Animals, Materials and Methods

### Animals and housing conditions

**Figure 1:**
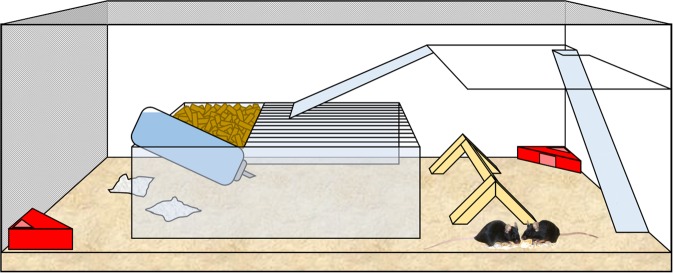
Super-enriched terrarium. Each terrarium (100 cm × 34 cm × 40.5 cm)^25^ included wire mesh on top and on one of the short sides, a second level that could be reached via two stairs and a passable standard polycarbonate cage type III equipped with food and water. Additionally, wood shavings, two semi-transparent red plastic houses, a wooden climbing frame and pressed cotton as nesting material were provided (photo of the two black mice: Dirk-Heinz Loddenkemper).

The study comprised 80 adult female Tph2 constitutive homozygous knockout (KO; n = 40) and wildtype (WT; n = 40) mice. They were derived from the internal stock of the Division of Molecular Psychiatry, University of Würzburg and transferred to the Department of Behavioural Biology in Münster, where the study was performed. During the experiment, the animals were kept in pairs in spacious and structured terraria (100 cm × 34 cm × 40.5 cm; “super-enriched terrarium”^25^; Fig. 1). Each terrarium included wire mesh on top and on one of the short sides, a second level that could be reached via two stairs and a passable standard polycarbonate cage type III equipped with standard mouse diet (Altromin 1324, Altromin GmbH, Lage, Germany) and tap water. Additionally, wood shavings as bedding material (Allspan, Höveler GmbH & Co. KG, Langenfeld, Germany), two semi-transparent red plastic houses (11.1 cm × 11.1 cm × 5.5 cm, Tecniplast Deutschland GmbH, Hohenpeißenberg, Germany), a wooden climbing frame and pressed cotton as nesting material were provided. The colony room was maintained at a temperature of about 22 °C, a relative humidity of about 50%, and a 12 h light-dark cycle. As all experimental animals were previously housed in groups of varying size and genotype composition (which might affect social behaviour), a “wash-out phase” preceded the experiment: for two weeks, each experimental mouse was housed together with two female mice of the C57BL/6J strain in a standard polycarbonate cage type III (37 cm × 21 cm × 15 cm). With this approach we aimed to standardize the housing conditions prior to the beginning of the experiment (i.e. no variation in group size, no variation in the cage-mates’ genotypes). C57BL/6J is the background strain of the Tph2 knockout mouse model. These mice express Tph2 and can thus produce brain serotonin.

All procedures complied with the regulations covering animal experimentation within the EU (European Communities Council DIRECTIVE 2010/63/EU), were conducted in accordance with the institutional and national guidelines for the care and use of laboratory animals, and were approved by the local government authorities (Amt für Gesundheit, Veterinär- und Lebensmittelangelegenheiten, Münster, reference number: 39.32.7.1).

### Experimental design

**Figure 2:**
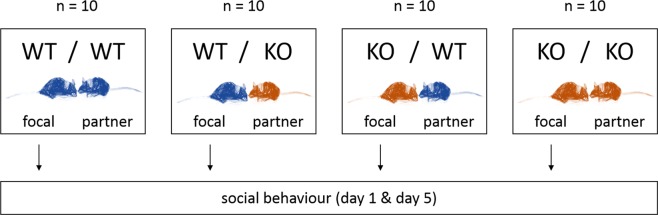
Experimental design. Female Tph2 WT and KO focal mice were housed together in pairs with each one unfamiliar female partner mouse of either the same or the other genotype. Social behaviour of the focal animal (arrows) was recorded for two hours directly after introduction on day 1 as well as on day 5 (original photo of the two mice: Dirk-Heinz Loddenkemper).

This study aimed to elucidate the effects of 5-HT deficiency caused by homozygous constitutive knockout of Tph2 on spontaneous social behaviour in a pair housing context. For this purpose, female Tph2 WT and KO mice were housed together in pairs with each one female mouse of either the same or the other genotype. As behaviour of two individuals in a cage is highly dependent, only behaviour of one mouse per terrarium was analysed (focal animal). The second mouse is referred to as “partner animal”. This approach thus resulted in four different combinations (focal animal/partner animal, n = 10 each): WT/WT, WT/KO, KO/WT and KO/KO (Fig. 2). Social behaviour of the focal animals was recorded during the first two hours after introduction on day 1 as well as during two hours on day 5. With this, we aimed to assess behaviour towards an unfamiliar individual (day 1) and towards a familiar individual (day 5). The 2 × 2 factorial design allowed analysing the behavioural data for an effect of the focal animal’s genotype (WT vs. KO), an effect of the partner animal’s genotype (WT vs. KO) as well as interactions between these factors. The decision to record only behaviour of one individual per cage was made to account for the dependencies of social behaviours between two individuals. Exclusively females were investigated as co-housing of unfamiliar male mice might lead to serious injuries due to their territoriality, especially as Tph2 knockout males have been found to be particularly aggressive^21,22^.

### Observation of social behaviour

Behaviour of the mice was filmed during the first two hours of the dark phase (i.e. the animals’ active period) on day 1 and day 5 using an infra-red camera system (EH1000H-4 Nano cameras, AVer Information Inc., Taiwan). To allow individual identification, mice were colour-marked on the tail one day prior to the introduction into the terraria.

Videos were analysed by an experienced observer (N.K.) and social behaviour was recorded using the software Observer XT 7.0 (Noldus Information Technology, Wageningen, The Netherlands). During analysis, the observer was blind to the mice’s genotype. Focal sampling and continuous recording^30^ were applied to record frequencies and durations of social behaviour. Based on previous work^5,15,25–29^, the following behaviours were defined and categorized: social interest (*approaching*, *facial/body sniffing*, *ano-genital sniffing*, *following*), aggressive behaviour (*chasing*, *attacking*, *escalated fighting*), defensive behaviour (*avoiding, fleeing, defensive upright posture*), threat behaviour (*tail rattling*), and other behaviour (*mounting*) (for definitions see Table 1). Please note that *defensive upright posture* and *tail rattling* were only measured qualitatively.

**Table 1:** Description of behavioural patterns.

Social interest	
*Approaching*:	The focal mouse moves directly towards the partner mouse until the distance between both is less than one body length (frequency).
*Facial/body sniffing*:	The focal mouse contacts the head/body of the partner mouse excluding the ano-genital region (duration).
*Ano-genital sniffing*:	The focal mouse contacts the ano-genital region of the partner mouse (duration).
*Following*:	The focal mouse locomotes after the partner mouse, while its head is directed to the latter’s backside. The maximum distance between the animals is one body length (duration).
**Aggressive behaviour**	
*Chasing*:	The focal mouse performs “*following*” at a fast running speed mostly subsequent to an agonistic interaction, while the partner mouse displays “*fleeing*”. The maximum distance between the animals is two body lengths (duration).
*Attacking*:	The focal animal contacts the body of the cohabitant with its mouth, making it react with winced movement of either single extremities, the tail or the whole body (not counted during escalated fighting) (frequency).
*Escalated fighting*:	Physical struggle between focal and partner mouse that usually involves kicking, wrestling and rolling over and over (active/passive individual not determinable) (frequency).
**Defensive behaviour**	
*Avoiding*:	The partner mouse performs “approaching” and the focal mouse moves away immediately (within 1 sec), thereby changing its speed or direction (frequency).
*Fleeing*:	The focal mouse moves away from the cohabitant at a fast running speed, mostly after the partner mouse has displayed an aggressive behaviour. Meanwhile it can perform leaps and jumps (duration).
*Defensive upright posture*:	The focal mouse rears up on its hind paws and keeps still, with the forepaws rigidly stretched out towards the partner mouse (only qualitative).
**Threat behaviour**	
*Tail rattling:*	The focal mouse makes fast waving movements with its tail (only qualitative).
**Other behaviour**	
*Mounting*:	The focal mouse places its forepaws on the back of the partner mouse and mostly performs pelvic thrusts (duration). Please note: *mounting* can be driven by sexual motivation but is also displayed in an agonistic context. As this could not be distinguished, it is listed as “other” behaviour.
(For descriptions of behavioural patterns see also)^5,15,25–29^.	

### Statistical analyses

The analysis of the behavioural data is split into two parts. As the data were clearly not normally distributed, non-parametric statistics were applied. As a first step to avoid the so-called inflation of the alpha, we implemented a global statistical test in which we analysed whether there was a general effect of the focal animal’s genotype to at least one of the behaviours performed by the focal animals. In the second part, we then implemented local descriptive tests in which we analysed the behavioural data for an effect of the focal animal’s genotype, the partner animal’s genotype and for an effect of interactions between these factors on each single focal animal’s behaviour. A rejection of the global test’s null hypothesis in the first part justified the use of local tests in the second part. Statistical tests were performed and graphs were created using the software R^31^. For a detailed description of the statistics, please see Supplementary Methods (M1).

## Results

**Figure 3:**
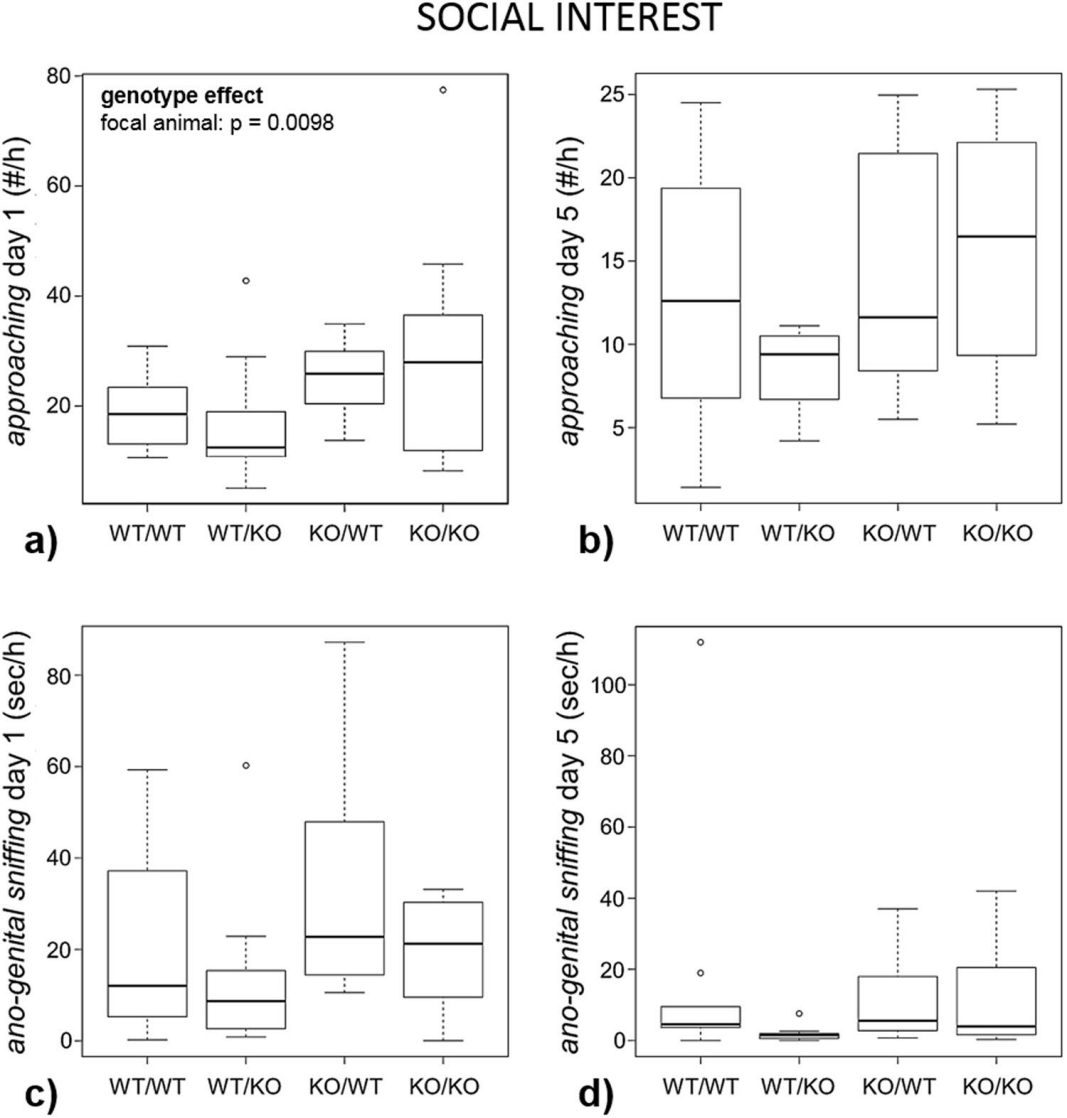
Social interest. Female Tph2 wildtype (WT) and knockout (KO) mice (focal animals) were housed together with a female of the same or the other genotype (partner animal), resulting in four combinations (focal animal/partner animal, n = 10 each): WT/WT, WT/KO, KO/WT and KO/KO. Behaviour was recorded on day 1 and day 5. Data are presented as boxplots with medians and 25–75% quartiles (box). The whiskers range from the minimum to the maximum of those observations with a distance from the box of less than 1.5 times the length of the box. Observations outside this area are represented as circles. Statistics: see Methods section; (**a**) *approaching* on day 1; (**b**) *approaching* on day 5; (**c**) *ano-genital sniffing* on day 1; (**d**) *ano-genital sniffing* on day 5.

**Figure 4:**
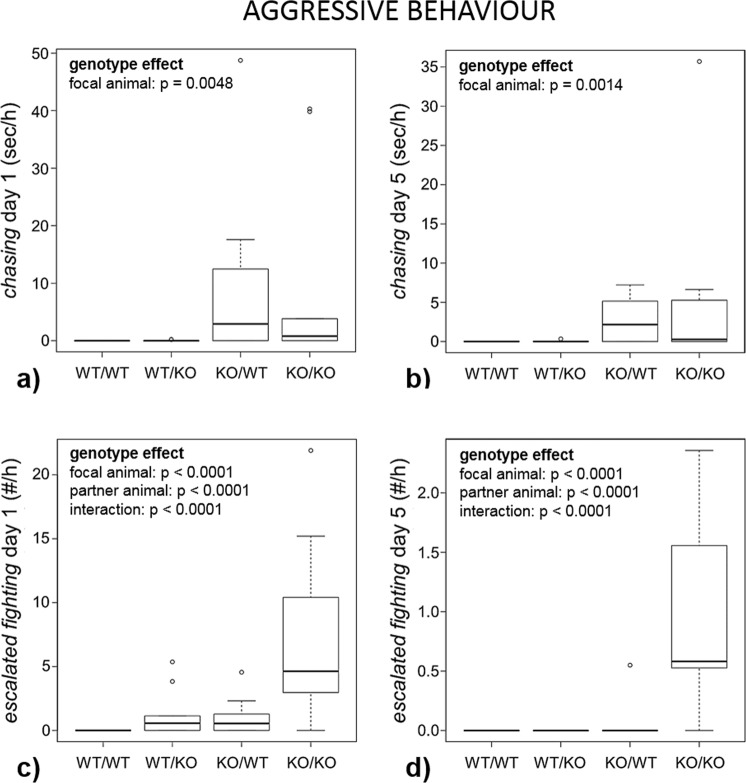
Aggressive behaviour. Female Tph2 wildtype (WT) and knockout (KO) mice (focal animals) were housed together with a female of the same or the other genotype (partner animal), resulting in four combinations (focal animal/partner animal, n = 10 each): WT/WT, WT/KO, KO/WT and KO/KO. Behaviour was recorded on day 1 and day 5. Data are presented as boxplots with medians and 25–75% quartiles (box). The whiskers range from the minimum to the maximum of those observations with a distance from the box of less than 1.5 times the length of the box. Observations outside this area are represented as circles. Statistics: see Methods section; (**a**) *chasing* on day 1; (**b**) *chasing* on day 5; (**c**) *escalated fighting* on day 1; (**d**) *escalated fighting* on day 5.

**Figure 5:**
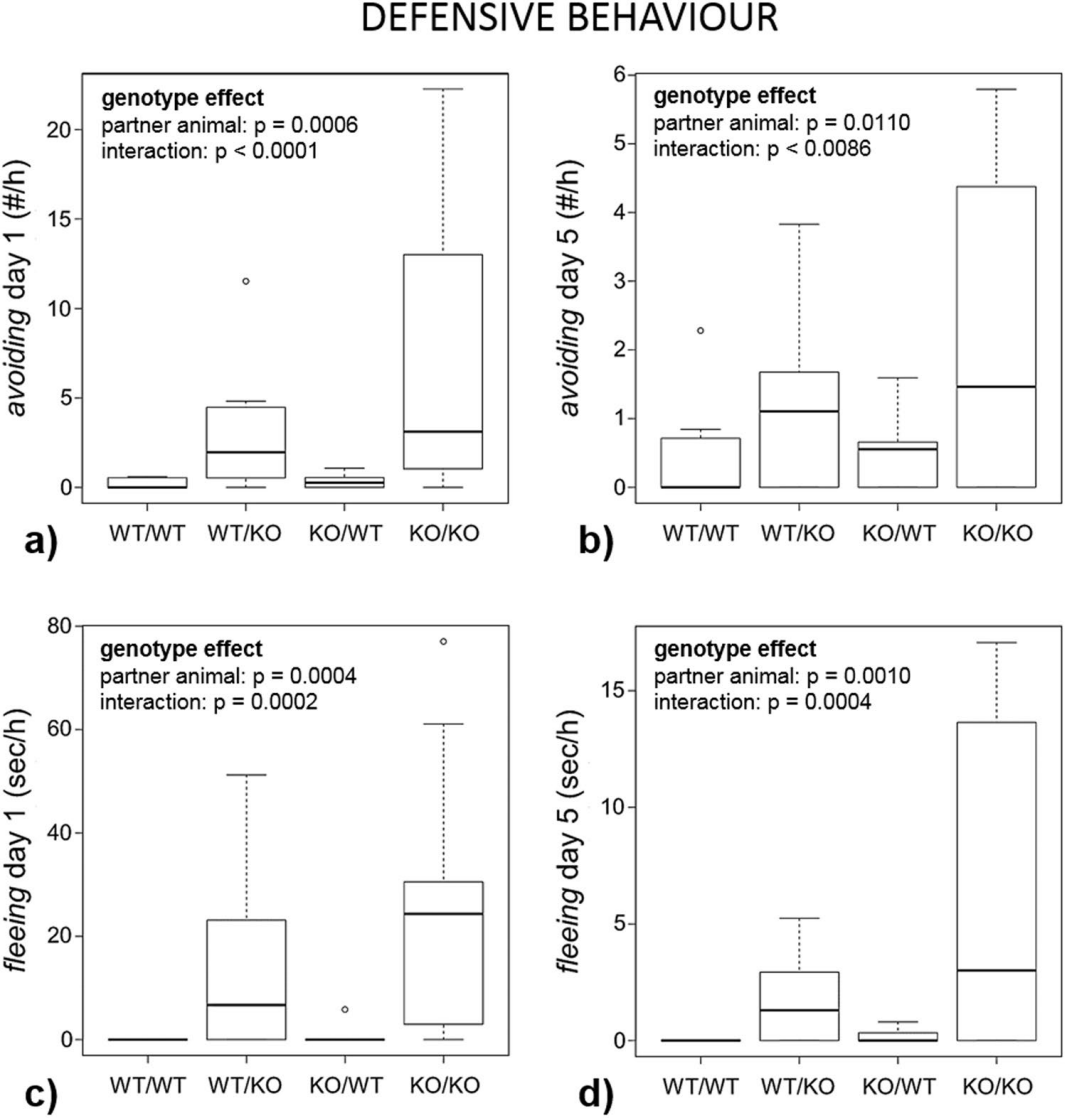
Defensive behaviour. Female Tph2 wildtype (WT) and knockout (KO) mice (focal animals) were housed together with a female of the same or the other genotype (partner animal), resulting in four combinations (focal animal/partner animal, n = 10 each): WT/WT, WT/KO, KO/WT and KO/KO. Behaviour was recorded on day 1 and day 5. Data are presented as boxplots with medians and 25–75% quartiles (box). The whiskers range from the minimum to the maximum of those observations with a distance from the box of less than 1.5 times the length of the box. Observations outside this area are represented as circles. Statistics: see Methods section; (**a**) *avoiding* on day 1; (**b**) *avoiding* on day 5; (**c**) *fleeing* on day 1; (**d**) *fleeing* on day 5.

All twelve defined behaviours of different behavioural systems including social exploration, aggressive behaviour, defensive behaviour and threat behaviour were shown at least once by a KO as well as a WT mouse. The permutation test revealed a significant global effect of the focal animal’s genotype on social behaviour on day 1 (p = 0.0155; Figs 3–5). If, instead of using a permutation test, we had used the approximate F-distribution of the ${T}^{2}$-statistics, we would get the same result: the global effect remained significant (p = 0.0454).

### Effects of the focal animal’s genotype

There was a pronounced difference between KO and WT females in terms of aggressive behaviours on both day 1 and day 5: KO females *attacked* their partners more frequently (day 1: p = 0.0230; day 5: p = 0.0040), *chased* them for longer durations (day 1: p = 0.0048; day 5: p = 0.0014) and engaged more often in *escalated fighting* (day 1 and 5: p < 0.0001; Fig. 4). Additionally, KO mice approached the partners more frequently on day 1 (p = 0.0098; day 5: p = 0.0622; Fig. 3). The focal animal’s genotype did overall not affect the frequency or duration of the other measured behaviours (Table 2).

**Table 2:** Results of the statistical tests (see Methods section) for effects of the focal animal’s genotype, the interaction partner’s genotype and interactions between these factors.

behavioural category	behaviour	day	WT/WT median *(Q1; Q3)*	WT/KO median *(Q1; Q3)*	KO/WT median *(Q1; Q3)*	KO/KO median *(Q1; Q3)*	focal animal’s genotype	partner animal’s genotype	interaction between focal and partner animals’ genotypes
social interest	*approaching* (#/hour)	1	18.5 *(12.7; 23.9)*	12.5 *(10.1; 21.5)*	25.8 *(19.7; 30;4)*	27.9 *(11.3; 38.8)*	**p = 0.0098**	p = 0.6604	p = 0.6816
		5	12.6 *(6.7; 20;5)*	9.4 *(6.3; 10.6)*	11.6 *(8; 22.1)*	16.5 *(9.2; 22.2)*	p = 0.0622	p = 0.4302	p = 0.4324
	*body/facial sniffing* (sec/hour)	1	23.1 *(6.7; 77.5)*	12.9 *(6.8; 39.8)*	34.2 *(8.8; 67.2)*	28.3 *(11.1; 41;7)*	p = 0.6332	p = 0.6566	p = 0.6306
		5	9.7 *(4.1; 22.1)*	4.1 *(2.1; 13.6)*	9.4 *(2.8; 15.5)*	8.1 *(5.1; 14.4)*	p = 0.4510	p = 0.3740	p = 0.3650
	*ano-genital sniffing* (sec/hour)	1	12.1 *(4.8; 42.4)*	8.7 *(2.4; 17.3)*	22.8 *(14.3; 55.6)*	21.3 *(8.8; 30.5)*	p = 0.1324	p = 0.0938	p = 0.0872
		5	4.5 *(3.2; 11.9)*	1.5 *(0.4; 2.1)*	5.6 *(2.4; 18.4)*	4 *(1.5; 20.7)*	p = 0.8694	p = 0.2006	p = 0.2162
	*following* (sec/hour)	1	11.5 *(1.9; 46)*	9.5 *(1; 36.8)*	28.3 *(20.3; 94)*	30.9 *(9.1; 40.6)*	p = 0.0790	p = 0.1176	p = 0.1058
		5	6.6 *(0.2; 13.2)*	0.9 *(0.1; 1.9)*	6.1 *(2.4; 29)*	1.6 *(1.2; 21.2)*	p = 0.0558	p = 0.1874	p = 0.1832
aggressive behaviour	*chasing* (sec/hour)	1	0 *(0; 0)*	0 *(0; 0.1)*	2.9 *(0; 13.7)*	0.8 *(0; 12.8)*	**p = 0.0048**	p = 0.9760	p = 0.9614
		5	0 *(0; 0)*	0 *(0; 0)*	2.2 *(0; 5.2)*	0.3 *(0; 5.6)*	**p = 0.0014**	p = 0.6082	p = 0.5940
	*attacking* (#/hour)	1	0 *(0; 0)*	0 *(0; 0.1)*	0.3 *(0; 2.9)*	1.3 *(0; 4.1)*	**p = 0.0230**	p = 0.8714	p = 0.8458
		5	0 *(0; 0)*	0 *(0; 0)*	0.9 *(0; 1.2)*	0 *(0; 2.2)*	**p = 0.0040**	p = 0.4412	p = 0.4262
	*escalated fighting* (#/hour)	1	0 *(0; 0)*	0.6 *(0; 1.8)*	0.6 *(0; 1.5)*	4.6 *(2.9; 11.6)*	**p < 0.0001**	**p < 0.0001**	**p < 0.0001**
		5	0 *(0; 0)*	0 *(0; 0)*	0 *(0; 0)*	0.6 *(0.4; 1.7)*	**p < 0.0001**	**p < 0.0001**	**p < 0.0001**
defensive behaviour	*avoiding* (#/hour)	1	0 *(0; 0.6)*	2 *(0.4; 4.6)*	0.3 *(0; 0.7)*	3.1 *(0.8; 13.1)*	p = 0.2292	**p = 0.0006**	**p < 0.0001**
		5	0 *(0; 0.8)*	1.1 *(0; 2.1)*	0.6 *(0; 0.8)*	1.5 *(0; 4.6)*	p = 0.2418	**p = 0.0110**	**p = 0.0086**
	*fleeing* (sec/hour)	1	0 *(0; 0)*	6.7 *(0; 26.2)*	0 *(0; 0)*	24.4 *(2.5; 38.2)*	p = 0.3852	**p = 0.0004**	**p = 0.0002**
		5	0 *(0; 0)*	1.3 *(0; 3.4)*	0 *(0; 0.4)*	3 *(0; 14.3)*	p = 0.1154	**p = 0.0010**	**p = 0.0004**
other	*mounting* (sec/hour)	1	0 *(0; 0)*	0 *(0; 0.6)*	0.4 *(0; 0.69)*	0.4 *(0; 30.5)*	p = 0.0776	p = 0.2974	p = 0.3020
		5	0 *(0; 0.5)*	0 *(0; 0)*	0 *(0; 0)*	0 *(0; 0)*	p = 0.2126	p = 0.2206	p = 0.2042

Q1 = 1. quartile, Q3 = 3. quartile; bold numbers indicate p-values below 0.05.

### Effects of the partner animal’s genotype

The genotype of the partner animal affected the amount of defensive behaviour displayed by the focal animal: on day 1 as well as day 5, females housed together with a KO *avoided* the latter more frequently (day 1: p = 0.0006; day 5: p = 0.0110) and displayed longer durations of *fleeing* (day 1: p = 0.0004; day 5: p = 0.0010; Fig. 5). Furthermore, females housed together with a KO more often engaged in *escalated fighting* (day 1 and 5: p < 0.0001). None of the other measured behaviours were affected by the genotype of the partner animal (Table 2).

### Interactions between the focal animal’s genotype and the partner animal’s genotype

There was an interaction between the focal animal’s genotype and the partner animal’s genotype concerning *avoiding* (day 1, p < 0.0001; day 5: p = 0.0086), *fleeing* (day 1: 0.0002; day 5: p = 0.0004) as well as *escalated fighting* (day 1 and 5: p < 0.0001; for the other behaviours see Table 2). Graphical examination indicates that in all these parameters the effect of the partner animal’s genotype (higher duration/frequencies of the behaviours displayed towards KO females) was more pronounced in KO than in WT mice.

## Discussion

The most outstanding result of this study was: while WT females were rather docile, KO individuals displayed considerable amounts of aggressive behaviours in a pair-housing context. This confirms our hypothesis and corresponds to the untypically high levels of aggression displayed by Tph2 KO females towards unfamiliar WT females when tested in a resident intruder paradigm^21,23^. Furthermore, Tph2 KO females in the present study performed aggressive behaviours not only directly after introduction into the terraria but still after several days of co-housing. This indicates that the higher aggressiveness of KO mice is not only shown in response to an unfamiliar intruder in a test situation but also spontaneously in the housing context and might thus represent a generalized form of aggression.

As outlined in the introduction, the “serotonin deficiency hypothesis” links lower 5-HT concentrations to higher aggressiveness. However, there is also contradicting evidence^32,33^ and a recent meta-analysis in humans found a rather weak negative correlation between brain 5-HT concentrations and aggression^3^. Nevertheless, our results correspond to a dampening effect of 5-HT on aggression and would thus be in line with several previous studies in rodents^10–16^.

The overwhelming majority of studies on the connection between 5-HT and aggression has been performed in male rodents. The few studies investigating females have mainly focussed on maternal aggression: 5-HT has been found to affect aggression in lactating female rats^16^ and mice^6,34^. Beyond that, our data indicate a role of serotonergic neurotransmission also in the regulation of female aggression outside of a reproductive context.

Yet, although this study could clearly link Tph2 knockout to higher aggression, it is still possible, that the described behavioural effects were not directly caused by the absence of serotonergic neurotransmission^35^. Constitutive Tph2 KO mice lack 5-HT throughout development and compensatory mechanisms as well as a disturbed tryptophan metabolism may also play a role. In a previous study, however, restored brain serotonin synthesis in male Tph2 KO mice by supplement of 5-HT’s immediate precursor (5-hydroxytryptophan) resulted in decreased aggression in a resident intruder paradigm^12^.

Beyond the higher aggression, social behaviour did not seem to be affected profoundly by 5-HT deficiency. First of all, the repertoire of social behaviours was exactly the same in both genotypes. Moreover, despite a higher rate of *approaching* on day 1, KO females did overall not differ from WT females concerning levels of social interest, defensive behaviour and mounting. Thus, our results do not support the notion of decreased social interest in female Tph2 KO mice that was found in a social interaction test^23^. This different outcome, however, can be explained by the fact that in the previous study only social exploration of non-aggressive KO females was analysed, which might have affected the results.

In line with our second hypothesis, the genotype of the partner animal had a considerable effect on defensive behaviour of the focal animal on both time points: corresponding to the higher aggression in KO females, *avoiding* as well as *fleeing* were more frequently displayed towards mice of this genotype. Additionally, focal animals were more often involved in *escalated fighting* when housed together with KO individuals. This finding highlights the fact that agonistic behaviour is not only influenced by internal factors, but additionally depends on situational conditions, in this case the genotype of the interaction partner. This has also been described in previous studies performed in male mice varying in brain 5-HT concentrations^15^.

As expected, some behaviours were also affected by an interaction between the genotypes. *Escalated fighting* was considerably more frequent in the KO/KO condition than in the mixed genotype condition and did never occur between WT females, thus further underlining the higher aggressiveness of KO females. Additionally, such an interaction also existed concerning defensive behaviours: the effect of the partner animal’s genotype on the focal animal’s behaviour were stronger in KO than in WT females. Thus, despite their higher aggressiveness, KO females were not only able to display *fleeing* and *avoiding* when meeting an aggressive opponent (particularly a KO) but they did so even more than WT females. As discussed by Angoa-Pérez and colleagues^21^, the higher aggressiveness in 5-HT deficient individuals might result from high impulsivity due to a lack of behavioural inhibition. In this context, our results indicate that this lack of inhibition in 5-HT deficient mice is not restricted to offensive aggression but also concerns defensive behaviours. This suggests a general increase of impulsivity and loss of behavioural control in KO females during agonistic social encounters.

## Conclusion

In this study, changes in social behaviour caused by Tph2 knockout were mainly related to aggressive behaviour: KO females displayed untypically high levels of aggressive behaviour even after several days of co-housing. Furthermore, they showed increased levels of defensive behaviours in response to aggressive partner animals. Thus, the results of this study on the one hand show, that 5-HT is not required to display the full range of social behaviours investigated in this study. On the other hand, they indicate an involvement of serotonergic neurotransmission in the control of female aggression and a prominent role in the fine-tuning of behaviour during agonistic social interactions. Future research will show, whether the observed behavioural effects are indeed directly caused by the lack of serotonin or by potential compensatory mechanisms.

## Supplementary information

Supplementary Information M1 and S1

## Data Availability

The data generated and analysed in this study are provided as electronic supplementary material.
