# Supplementary material for: Brain serotonin deficiency affects female aggression
Source: Sci Rep. 2019 Feb 4;9:1366. doi: 10.1038/s41598-018-37613-4 (PMC6361930; doi:10.1038/s41598-018-37613-4)
Supplement: Supplementary file 1 — Supplementary Information M1 and S1 [file 41598_2018_37613_MOESM1_ESM.pdf]

Brain serotonin deficiency affects female aggression

Niklas Kästner[1,2], S. Helene Richter[1,2], Sarah Urbanik[3], Joachim Kunert[3], Jonas Waider[4], Klaus-Peter Lesch[4,5,6], Sylvia Kaiser[1,2], Norbert Sachser[1,2]*

Affiliations

[1] Department of Behavioural Biology, University of Münster, Germany

[2] Münster Graduate School of Evolution, University of Münster, Germany

[3] Department of Mathematical Statistics and Applications in Science, Technical University of Dortmund, Germany

[4] Division of Molecular Psychiatry, Center of Mental Health, University of Würzburg, Würzburg, Germany

[5] Laboratory of Psychiatric Neurobiology, Institute of Molecular Medicine, I.M. Sechenov First Moscow State Medical University, Moscow, Russia

[6] Department of Neuroscience, School for Mental Health and Neuroscience (MHeNS), Maastricht University, Maastricht, The Netherlands

Supplementary Methods M1

Detailed statistical analyses

Global Test: We tested the hypothesis

> H_0: There is no effect of the focal animal's genotype to the focal animal's behaviours *approaching*, *facial/body sniffing*, *ano-genital sniffing*, *following, chasing*, *attacking*, *escalated fighting*, *avoiding, fleeing* and *mounting*.

against the alternative

> H_1: There is an effect of the focal animal's genotype to at least one of the focal animal's behaviours *approaching*, *facial/body sniffing*, *ano-genital sniffing*, *following, chasing*, *attacking*, *escalated fighting*, *avoiding, fleeing* or *mounting*.

A standard method to compare two vectors of means is Hotelling's T^2- test with test statistic

$$T^2 = \frac{n_1 * n_2}{n_1 + n_2} (\overline{\boldsymbol{x_1}} - \overline{\boldsymbol{x_2}})^T \boldsymbol{S}^{-1} (\overline{\boldsymbol{x_1}} - \overline{\boldsymbol{x_2}}) \frac{n_1 + n_2 - p - 1}{p * (n_1 + n_2 - 1)}$$

where $n_1 = 20$ is the number of animals with genotype WT, $n_2 = 20$ is the number of animals with genotype KO, $p = 10$ is the number of variables (behaviours), $\overline{\boldsymbol{x}}$ describes the sample mean and $\boldsymbol{S}$ describes the sample covariance. Under H_0, the test statistic T^2 approximately follows an F distribution with $p = 10$ and $n_1 + n_2 - p - 1 =$ 29 degrees of freedom[1]. The behavioural data clearly were not normally distributed. This suggests that the assumption of an F distribution may not be appropriate. We therefore applied a permutation test at significance level 5% with $10{,}000$ permutations using the test statistic T^2.

Further Analysis (descriptive): We further analysed the behavioural data for an effect of the focal animal's genotype, the partner animal's genotype and for an effect of interactions between these factors on each single focal animal's behaviour. For each effect and each behaviour, we applied a permutation test at level 5%. Note that (to avoid the so-called inflation of the alpha-level) an interpretation of the results is valid only in a descriptive sense. For a

fixed behaviour, let $x_{klj}^{(d)}$ describe the behaviour of focal animal $j \in \{1, \dots, 10\}$ in group (k, l), $k, l \in \{1 = WT, 2 = KO\}$, at day $d \in \{1,5\}$. We assume that the variables satisfy the following identities

$$E\left(x_{WT,WT,j}^{d}\right) = \mu + \alpha + \beta + \gamma \qquad E\left(x_{WT,KO,j}^{d}\right) = \mu + \alpha - \beta - \gamma$$

$$E\left(x_{KO,WT,j}^{d}\right) = \mu - \alpha + \beta - \gamma \qquad E\left(x_{KO,KO,j}^{d}\right) = \mu - \alpha - \beta + \gamma$$

where α describes the effect of the focal animal's genotype, β describes the effect of the partner animal's genotype and γ describes the effect of interactions between focal and partner animal's genotype. Then each group's sample mean yields an estimator for

$$\mu + (-1)^{k+1}\alpha + (-1)^{l+1}\beta + (-1)^{k+l}\gamma$$

For that reason, the application of a permutation test requires an appropriate adjustment of the observations such that a certain mean coincides with a certain effect. Therefore, for each permutation, we first adjusted the observations and then calculated the test statistic.

In the case of the focal animal's genotype, we tested the hypothesis $H_0: \alpha = 0$ against the alternative $H_1: \alpha \neq 0$, in the case of the partner animal's genotype, we tested the hypothesis $H_0: \beta = 0$ against $H_1: \beta \neq 0$, and in the case of interactions we tested the hypothesis $H_0: \gamma = 0$ against $H_1: \gamma \neq 0$. In each case, we use an adjusted t-test statistic.

Reference:

1. Srivastava & Carter (1983). An introduction to applied multivariate statistics. North Holland, New York.

Supplementary Dataset S1

Supplementary Dataset

				approaching	body/facial sniffing	ano-genital sniffing	following	mounting	chasing	attacking	fleeing	avoiding	escalated fighting
Pair	Genotype focal animal (1 = WT, 2 = KO)	Genotype partner animal (1 = WT, 2 = KO)	Day	#/hour	sec/hour	sec/hour	sec/hour	sec/hour	sec/hour	#/hour	sec/hour	#/hour	#/hour
1	1	1	1	30.8827771	30.5739493	37.1871233	43.5553649	0	0	0	0	0.53246167	0
2	1	1	1	23.3805778	15.7030485	6.63899663	5.29597274	0	0	0	0	0.54373437	0
3	1	1	1	18.9015766	42.3796258	29.5895589	30.7465645	0	0	0	0	0	0
4	1	1	1	18.1030543	3.84423682	3.10414137	1.77303444	0	0	0	0	0	0
5	1	1	1	20.7048738	105.698381	59.2922202	53.3586393	0.56121105	0	0	0	0	0
6	1	1	1	11.7202952	192.59375	58.1150835	99.3705225	0	0	0	0	0.58601476	0
7	1	1	1	25.3103531	68.1330601	15.3609738	14.2159817	0	0	0	0	0.60262746	0
8	1	1	1	10.6271494	15.6750454	8.74968635	8.75559032	0	0	0	0	0	0
9	1	1	1	13.0788538	7.13081855	0.23883124	0.89277393	0	0	0	0	0	0
10	1	1	1	17.8074102	5.33682686	5.29905357	1.88326853	0	0	0	0	0	0
11	2	1	1	34.91344	35.4075995	78.4263289	154.12941	0.51027335	2.66953534	0	0	1.07425969	1.07425969
12	2	1	1	20.3943208	5.60568224	16.8501186	20.8132312	82.4647119	0	0	0	0	0
13	2	1	1	29.0308809	66.9588729	24.5567098	21.5682368	0.51800199	12.4605095	2.84616479	0	0	4.55386367
14	2	1	1	13.7723212	49.1195131	14.4503431	18.0470378	0.19599072	0	0	0	1.05940932	0
15	2	1	1	31.7171471	67.8573946	87.1875541	269.56115	0.61127593	17.5885998	2.88337701	0	0	2.30670161
16	2	1	1	22.5480521	82.9328355	20.9971861	35.0154751	0	0	0	5.8294964	0.54995249	0.54995249
17	2	1	1	23.9593072	32.9668658	47.9357282	73.9829179	0	2.44727209	0.5704597	0	0	0
18	2	1	1	29.9306395	9.83253347	36.8210548	40.3235764	0.91702385	48.7359965	30.5674616	0	0	1.27364423
19	2	1	1	27.7057636	2.29251612	13.9235043	21.5127105	0.05975753	6.04094296	1.6297508	0	0.54325027	0
20	2	1	1	17.4124127	12.6403233	10.567158	18.8543781	0	3.16144118	0	0	0.5441379	0.5441379
21	1	2	1	11.0039023	2.16934074	0.86983228	0	0	0.19387828	0	23.1239147	11.5278977	1.0479907
22	1	2	1	13.6841545	11.7628992	3.96293115	6.01008066	0	0	0	18.6049765	2.18946472	3.83156326
23	1	2	1	28.9484476	204.103925	22.8750633	45.6748607	3.69961161	0	0	0	1.73690686	0
24	1	2	1	11.2598356	8.60036967	4.0910736	1.23858192	0	0	0	35.2325618	4.82564383	5.36182648
25	1	2	1	5.03952293	14.0546695	13.6235103	12.9347755	0	0	0	51.2351497	4.47957593	0.55994699
26	1	2	1	42.7706502	59.3949266	60.2447119	58.4944919	0.38268476	0	0	0	0	0.56277171
27	1	2	1	17.7807845	21.1699098	15.3776845	33.7834905	1.16922128	0	0	0	0.53881165	0
28	1	2	1	18.9628809	33.2446392	13.3281963	19.2825409	0.40634745	0.23297254	1.0835932	0	0	0
29	1	2	1	7.8029326	6.56003691	1.41010139	0.41244072	0	0	0	2.37989444	1.67205699	0
30	1	2	1	10.8100761	6.81603747	2.67407146	1.72961218	0	0	0.56895138	11.0035196	3.98265963	1.13790275
31	2	2	1	34.5053173	40.0864154	32.4076131	45.4703403	3.32455994	0.91466476	1.64311035	22.4722725	3.83392414	21.908138
32	2	2	1	36.305071	28.9248461	26.2426058	29.2824781	157.200957	0	0	26.2263498	13.0048015	0
33	2	2	1	77.4618108	32.6684428	21.5171697	45.9983294	0	39.8336603	24.2068159	0	0	7.53100938
34	2	2	1	45.8004673	27.7301011	30.3271958	37.8009993	0.83794037	40.2992066	10.929657	0.85875876	1.04091971	10.4091971
35	2	2	1	36.4917394	46.4397049	21.0224151	32.4776481	0	3.83427697	1.58659737	2.99866902	2.11546315	3.17319473
36	2	2	1	21.3554094	117.24713	33.1364769	38.9736221	112.026918	0	0	30.5441675	0	2.96602908
37	2	2	1	11.9268882	12.0640474	9.5772912	6.6552036	0	0.67386918	1.78903323	6.25565285	2.38537764	5.96344409
38	2	2	1	19.5509025	5.92500962	0.06516967	0	0	0.97211432	1.08616125	61.0965703	22.2663056	15.2062575
39	2	2	1	9.21318445	8.30338249	6.76593233	9.96751393	0	0	0	26.7988503	9.21318445	2.87912014
40	2	2	1	8.23450655	20.2513964	20.4160866	23.012701	1.16381026	0	0	77.0530226	13.1752105	3.29380262
1	1	1	5	6.78261048	3.73043577	4.47035691	0.41928865	0	0	0	0	0	0
2	1	1	5	6.34854699	2.08091262	3.66804937	0	0	0	0	0	0	0
3	1	1	5	8.01604694	5.67135321	111.937415	12.3580724	0	0	0	0	0	0
4	1	1	5	10.4230356	4.16921424	1.96811437	0.31269107	0	0	0	0	0	0
5	1	1	5	24.5057801	19.3379435	3.762358	2.55148416	0	0	0	0	0	0
6	1	1	5	23.955868	53.9166735	18.9810327	31.3742017	11.5068019	0	0	0	0	0
7	1	1	5	15.9634614	13.6943694	4.61800134	10.7069216	0	0	0	0	2.28049449	0
8	1	1	5	1.42410697	5.04133866	0	0	0	0	0	0	0.71205348	0
9	1	1	5	19.374086	21.4715414	9.51014917	17.4029833	1.97952618	0	0	0	0.84235156	0
10	1	1	5	14.8126681	23.7968732	8.77811589	11.6955675	0	0	0	0	0	0
11	2	1	5	24.9622261	15.2617889	37.0079515	51.4454064	0	2.78067588	1.16103377	0	0	0
12	2	1	5	23.8610878	16.247736	19.6493283	28.0062581	0	0	0	0	0.55490902	0
13	2	1	5	10.6257214	9.71973883	4.99968154	2.4886558	0	5.46385779	0.55924849	0.32995661	1.11849699	0
14	2	1	5	10.0794716	9.01847461	6.18561259	7.08746005	0	0	0	0.79574776	1.59149552	0
15	2	1	5	8.41190284	2.84202146	4.19393442	5.16731175	0	1.53817652	1.20170041	0	0.6008502	0
16	2	1	5	5.50750934	2.93550248	0.69394618	0	0	0	0	0.61684105	0.55075093	0
17	2	1	5	21.4554562	13.3573969	17.9895748	31.847599	0	7.22333693	1.65041971	0	0.5501399	0.5501399
18	2	1	5	6.56133188	2.60663821	2.78558363	1.93857533	0	5.13573341	1.19296943	0	0	0
19	2	1	5	15.7944672	18.2294475	17.1040918	26.56761	0	3.23786577	0.6581028	0	0.6581028	0
20	2	1	5	12.6442714	0.97305915	1.05002428	3.1830579	0	0.86860647	1.09950186	0	0	0
21	1	2	5	9.80476973	5.31187819	1.79946362	1.91481385	0	0	0	5.23690054	1.15350232	0
22	1	2	5	8.99899511	2.99966504	0	0.63592899	0	0	0	0.2999665	0	0
23	1	2	5	11.1162766	26.16648	1.88359131	0.14204131	0	0	0	2.92728617	1.23514184	0
24	1	2	5	9.80355623	4.5044761	1.21254511	1.87299522	0	0	0	2.2909363	0	0
25	1	2	5	7.11024681	9.38005637	2.57609711	1.34000805	0	0	0	4.9334174	3.82859443	0
26	1	2	5	11.0536165	27.1744435	7.56881846	7.65026616	0	0	0	0	0	0
27	1	2	5	4.21512351	2.07067943	1.18023458	0	0	0	0	0	1.05378088	0
28	1	2	5	10.5073595	3.77214207	1.95436887	0.62518789	0	0.3362355	0	0	0	0
29	1	2	5	5.15958074	1.22110077	0.56755388	0	0	0	0	0	3.43972049	0
30	1	2	5	6.70322406	2.0891715	0	1.13954809	0	0	0	2.51370902	1.67580602	0
31	2	2	5	17.5588481	20.6741276	21.2745269	22.8208384	0	5.26199029	1.13282891	0	0	2.26565782
32	2	2	5	11.0599402	16.826909	8.33708825	1.34299274	0	0	0	6.76763007	5.79330201	0.52666382
33	2	2	5	25.3162014	13.6139164	42.0197277	54.3523344	0	6.6287115	1.54997151	0	0.51665717	0
34	2	2	5	22.3062148	6.961614	20.5009676	20.6047174	0	35.7003186	12.4499803	0	0	1.55624754
35	2	2	5	17.0806488	7.30344983	1.18386566	1.53136851	0	0.50063971	4.12291523	0	1.76696367	2.35595156
36	2	2	5	9.34767509	4.761472	1.64168544	0.40311849	0	0	0	13.635921	5.25806724	0.58422969
37	2	2	5	5.21349587	5.27142361	0.30701698	1.55825599	0	0	0	6.00131303	1.15855464	0.57927732
38	2	2	5	22.1226637	11.5775274	3.66604142	5.50959673	0	4.47720576	0	0	0	1.05346018
39	2	2	5	15.8732813	4.72366959	3.21844463	1.49427786	0	0	0	17.0665142	4.37883623	0.54735453
40	2	2	5	8.85690199	8.94547101	4.23950375	0.64950615	0	0	0	16.231749	2.95230066	0
